# Disaster Risk Reduction, Climate Change Adaptation and Their Linkages with Sustainable Development over the Past 30 Years: A Review

**DOI:** 10.1007/s13753-023-00472-3

**Published:** 2023-02-07

**Authors:** Jiahong Wen, Chengcheng Wan, Qian Ye, Jianping Yan, Weijiang Li

**Affiliations:** 1 School of Environment and Geographical Sciences, Shanghai Normal University, Shanghai, 200234 China; 2 Integrated Risk Governance Project, Beijing, 100875 China; 3 Rodel Risk Solutions Inc., Toronto, ON M1W1J3 Canada

**Keywords:** Climate change adaptation, Disaster risk reduction, Resilience, Sustainable development goals, United Nations

## Abstract

The severe damage and impacts caused by extreme events in a changing climate will not only make the sustainable development goals difficult to achieve, but also erode the hard-won development gains of the past. This article reviews the major impacts and challenges of disaster and climate change risks on sustainable development, and summarizes the courses and linkages of disaster risk reduction (DRR), climate change adaptation (CCA), and sustainable development over the past 30 years. Our findings show that the conceptual development of DRR actions has gone through three general phases: disaster management in the 1990s, risk management in the 2000s, and resilient management and development in the 2010s. Gradually, CCA has been widely implemented to overcome the adverse effects of climate change. A framework is proposed for tackling climate change and disaster risks in the context of resilient, sustainable development, indicating that CCA is not a subset of DRR while they have similarities and differences in their scope and emphasis. It is crucial to transform governance mechanisms at different levels, so as to integrate CCA and DRR to reduce disaster and climate change risks, and achieve safe growth and a resilient future in the era of the Anthropocene.

## Introduction

Frequent disasters triggered by natural hazards around the world have caused huge losses of life and property to human society (CRED and UNDRR 2020). Climate change is further exacerbating disaster risks, increasing the frequency and severity of disaster damage and losses, and seriously hindering our efforts to achieve the sustainable development goals (SDGs) (IPCC 2022). Disaster risk reduction (DRR) and climate change adaptation (CCA) have become significant common challenges facing the international community in the era of the Anthropocene.

In December 1989, the United Nations adopted a historical resolution, declaring that the International Decade for Natural Disaster Reduction (IDNDR) would be launched on 1 January 1990 (United Nations 1989). Since then, international disaster reduction efforts have been developing vigorously for more than 30 years. Global actions on climate change mitigation and adaptation also go back more than 30 years. In November 1988, the World Meteorological Organization and the United Nations Environment Programme jointly established the Intergovernmental Panel on Climate Change (IPCC).^1^ In December 1990, the 45th session of the United Nations General Assembly endorsed resolution 45/212, deciding to establish the Intergovernmental Negotiating Committee for the United Nations Framework Convention on Climate Change (UNFCCC) (United Nations 1992a) with the participation of all member states of the United Nations, to negotiate international conventions on climate change, which was finally adopted in May 1992 (United Nations 1992a). Since then DRR and CCA have become the core themes for international sustainable development.

Some previous studies have considered that CCA is a subset of disaster risk reduction and one of many processes within disaster risk reduction (Kelman 2015; Kelman et al. 2015). This may not be the case, however, in many ways, disaster risk reduction and CCA have overlapping aims and involve similar kinds of intervention (Twigg 2015; Islam et al. 2020). Therefore, many studies have suggested that addressing CCA and DRR together could be more beneficial (Clegg et al. 2019), and various studies have also explored ways and barriers of integrating DRR with CCA, as well as mainstreaming both into development (Mitchell et al. 2010; Florano 2015; Twigg 2015; Hore et al. 2018; Mal et al. 2018; Gabriel et al. 2021).

In the context that more than three years of the COVID-19 pandemic have affected all dimensions of social-ecological systems, and the proposed 2015−2030 sustainable development agenda has already been implemented halfway, the three main objectives of this study are to: (1) review the challenges, impacts, and risks of climate change and extreme events; (2) summarize the agenda and concept evolution of international DRR, CCA, and sustainable development since 1990; and (3) discuss the governance mechanisms and practices of integration of DRR and CCA—and their linkages with sustainable and resilient development—employed by the members of the international community over the past 30 years. Such work could help us find ways to achieve the goals set by the United Nations’ Sendai Framework for Disaster Risk Reduction 2015−2030 (United Nations 2015a), the Paris Agreement (United Nations 2015b), and the 2030 Agenda for Sustainable Development (United Nations 2015c).

## Disaster Risk Reduction and Sustainable Development

From 2000 to 2019, 7,348 disaster events were recorded worldwide by EM-DAT (The International Disaster Database at the Centre for Research on the Epidemiology of Disasters) (CRED and UNDRR 2020). These disasters claimed approximately 1.23 million lives, an annual average of 60,000 lost lives, and affected a total of over 4 billion people (many on more than one occasion) (CRED and UNDRR 2020). These disasters also led to approximately USD 2.97 trillion in direct economic losses worldwide. If the expected annual losses induced by natural hazards were shared equally among the world’s population, it would be equivalent to an annual loss of almost USD 70 for each individual of working age, or two months’ income for people living below the poverty line (UNISDR 2015). Clearly, sustainable development cannot be achieved without taking account of disaster risk reduction (UNDP 2004; UNDRR 2022). To do so, however, there are three major obstacles that need to be addressed.

First, there is still a lack of scientific and technological capabilities (including risk monitoring, risk assessment, early warning, and so on) and risk governance mechanisms to reduce the loss of life and property caused by very large-scale disasters globally. The 2008 Wenchuan Earthquake in China caused a total of 87,150 deaths and missing persons; in 2010, the Haiti Earthquake killed 222,500 people; the 2015/2016 droughts in India affected 330 million people; the direct economic losses caused by the 2011 East Japan Earthquake and Tsunami were as high as USD 210 billion (CRED and UNDRR 2020).

Second, EM-DAT does not record many small-scale but recurring disasters caused by extensive risks (minor but recurrent disaster risks) (UNISDR 2015), as well as indirect losses. From 2005 to 2014, direct economic losses due to extensive risks in 85 countries and territories were equivalent to a total of USD 94 billion (UNISDR 2015). Extensive risks are responsible for most disaster morbidity and displacement, and represent an ongoing erosion of development assets, such as houses, schools, health facilities, and local infrastructures. However, the cost of extensive risk is not visible and tends to be underestimated, as it is usually absorbed by low-income households and communities and small businesses. In addition, better recording and sharing of disaster information is needed for disaster loss accounting, forensics, and risk modeling (De Groeve et al. 2013; De Groeve et al. 2015; Hallegatte 2015; Khadka 2022; UNDRR 2022).

Third, in today’s crowded and interconnected world, indirect, cascading impacts can also be significant, and disaster impacts increasingly cascade across geographies and sectors (UNDRR 2022). Indirect losses, including output losses (such as business interruptions, supply-chain disruptions, and lost production due to capital damages), and macroeconomic feedbacks, may extend over a longer period of time than the event, and affect a larger spatial scale or different economic sectors (Hallegatte 2015). Therefore, indirect, cascading impacts may cause more serious harm to socioeconomic development in a region or society (Khadka 2022; UNDRR 2022).

## Climate Change Risks and Sustainable Development

The best estimate of total human-caused global surface temperature increase from 1850–1900 to 2010–2019 is around 1.1 °C, and each of the last four decades has been successively warmer than any decade that preceded it since 1850 (IPCC 2021; WMO 2021). If the temperature continues to rise at the current rate, global warming could reach 1.5 °C between 2030 and 2052 (IPCC 2018). Increasing risks associated with health, livelihoods, food security, water supply, human security, and economic growth are all expected in a rapidly changing climate (Carleton and Hsiang 2016; IPCC 2018). The Sixth Assessment Report of the Intergovernmental Panel on Climate Change (IPCC AR6) has identified over 130 key risks (KRs) that may become severe under particular conditions of climate hazards, exposure, and vulnerability. These key risks are represented in eight so-called Representative Key Risk (RKR) clusters of key risks relating to low-lying coastal systems; terrestrial and ocean ecosystems; critical physical infrastructure, networks, and services; living standards; human health; food security; water security; and peace and mobility (IPCC 2022). The international scientific community has warned that without quick actions on the following three urgent issues, the severe damage and impacts of climate change and extreme events will not only put the achievement of the SDGs out of reach but also erode the hard-won development gains of the past.

The first issue is that as human-induced climate change, including more frequent and intense extreme events, has affected and will continue to threaten the lives and livelihoods of millions to billions of people, the challenges of how to significantly reduce the emerging risks of climate change are enormous ((IPCC 2018, 2022; Rising et al. 2022). Currently, climate-related disasters account for more than 80% of disasters caused by natural hazards (UNDRR 2021). Around the world 3.3−3.6 billion people live in areas of high vulnerability to climate change (IPCC 2022).

The second issue is that under higher warming scenarios (for example, 3−4 °C) it is almost certain that Planet Earth will cross tipping points, leading to irreversible changes in ecosystems or climate patterns, which will significantly limit our ability to adapt (Steffen et al. 2018; Lenton et al. 2019; Ritchie et al. 2021). The challenges of how to address the adaptation limits that are already being confronted across the world will only increase (Future Earth et al. 2022). For example, in high-emission scenarios, week-long heat extremes that break records by three or more standard deviations are two to seven times more probable in 2021–2050 and three to 21 times more probable in 2051–2080, compared to the last three decades (Fischer et al. 2021). Building codes in many areas have to be modified and even redesigned.

The third issue is the lack of scientific research to better understand the mechanisms of systemic risks caused by climate change in the context of deep uncertainty. For example, record-shattering extremes—nearly impossible in the absence of warming—are likely to occur in the coming decades (Fischer et al. 2021), which may lead to the emergence of systemic risks with large-scale, non-linear, and cascading consequences in socioeconomic systems (Helbing 2012; Renn et al. 2019). Deep uncertainty is mainly reflected in three aspects, including uncertain scenarios of climate change, uncertain consequences of decision making, and uncertain schemes of decision making. Due to the deep uncertainty of the changes, over- or under-adaptation can occur, leading policymakers and planners to make suboptimal decisions (Linstone 2004; Kwakkel et al. 2016; Marchau et al. 2019; Webber and Samaras 2022).

## Agenda and Evolution of International Disaster Risk Reduction, Climate Change Adaptation, and Sustainable Development

**Fig. 1:**
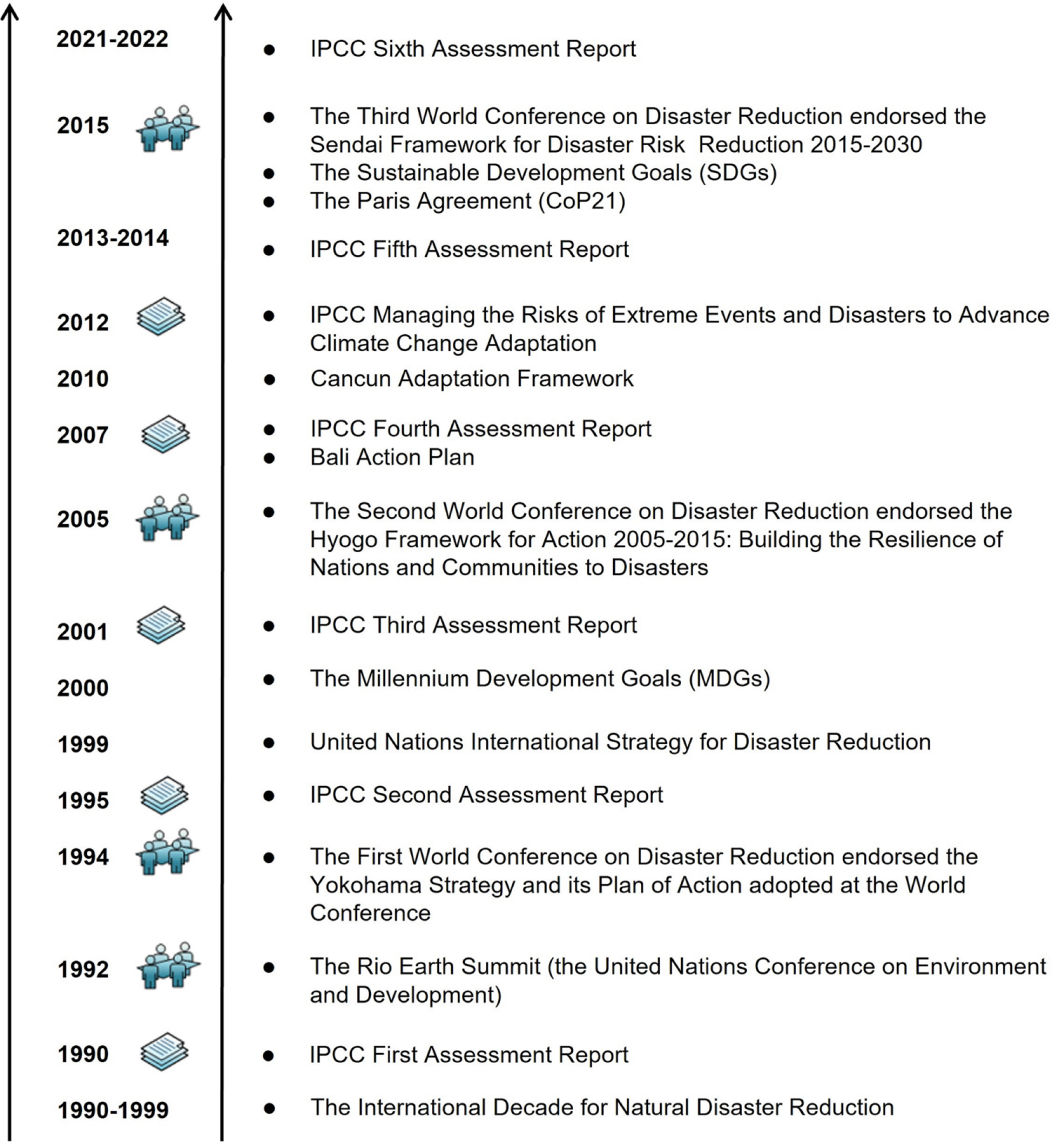
Important events of disaster risk reduction (DRR), climate change adaptation (CCA), and sustainable development since 1990. *IPCC:* Intergovernmental Panel on Climate Change. *Source* Modified from Mal et al. (2018)

A landmark year for DRR, CCA, and sustainable development was 2015 because three important events occurred in that year—the Sendai Framework for Disaster Risk Reduction 2015−2030, the Sustainable Development Goals (SDGs), and the Paris Agreement under the UNFCCC (United Nations 2015a; United Nations 2015b; United Nations 2015c) were adopted by the international community. Looking back in history can help us understand the governance of international DRR and CCA, and their important processes and context (Fig. 1).

### International Disaster Risk Reduction Action Framework and Concept Evolution

In 1987, the 42nd session of the United Nations General Assembly passed a resolution and decided to designate the 1990s as the International Decade for Natural Disaster Reduction (IDNDR) (United Nations 1987), calling on governments from all over the world to actively participate in and support this action. The main goal of the IDNDR was to minimize the losses of life and property, as well as the impacts and damage to the economy and society caused by disasters. In 1999, the United Nations International Strategy for Disaster Reduction (UNISDR) and the UNISDR Secretariat were established as the successor arrangements for the IDNDR to be responsible for the implementation of DRR plans and strategies among UN member states, with a view to further strengthening international disaster reduction efforts. In 2019, the Secretariat changed its name to the UN Office for Disaster Risk Reduction (UNDRR).^2^

The First World Conference on Natural Disaster Reduction held at Yokohama, Japan in 1994 adopted the Yokohama Strategy and Plan of Action for a Safer World (IDNDR 1994), reiterating the focus of the IDNDR. The Yokohama Plan of Action urged the incorporation of disaster prevention, preparedness, early warning, recovery, local capacity building, and improvement of disaster response mechanisms into national policies in order to reduce the impacts of disasters.

**Fig. 2:**
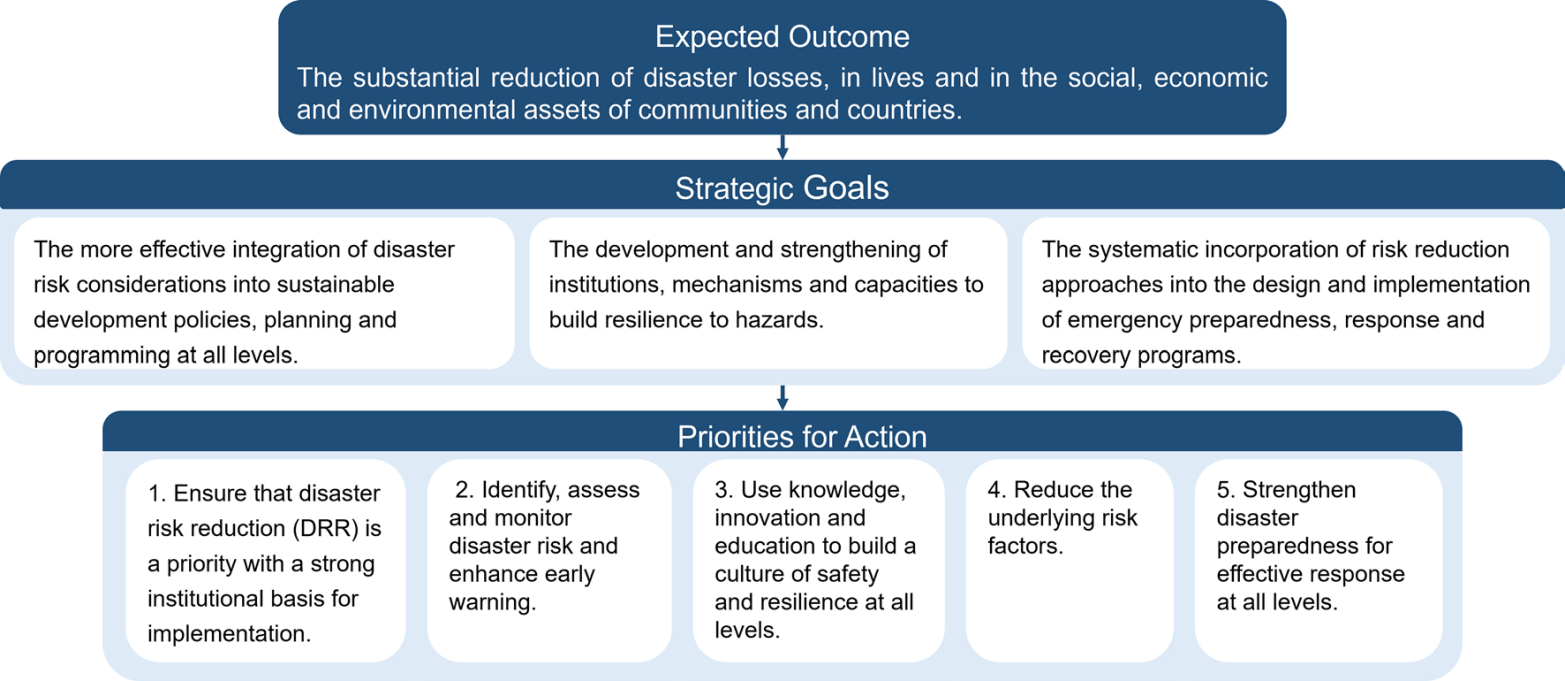
The Hyogo Framework for Action 2005−2015: Expected outcome, strategic goals, and priorities for action (United Nations 2005)

In 2005, the Second World Conference on Natural Disaster Reduction held at Kobe, Hyogo, Japan, adopted the Hyogo Declaration and the Hyogo Framework for Action 2005−2015: Building the Resilience of Nations and Communities to Disasters (United Nations 2005). The goals of the Hyogo Framework were to substantially reduce the loss of human, socioeconomic, and environmental assets of communities and countries from disasters by 2015 by integrating DRR into strategies and planning processes, and by promoting the effective role of local knowledge, resilience building, and climate adaptation. The action framework includes an expected outcome, three strategic goals, and five priorities for actions (Fig. 2).

**Fig. 3:**
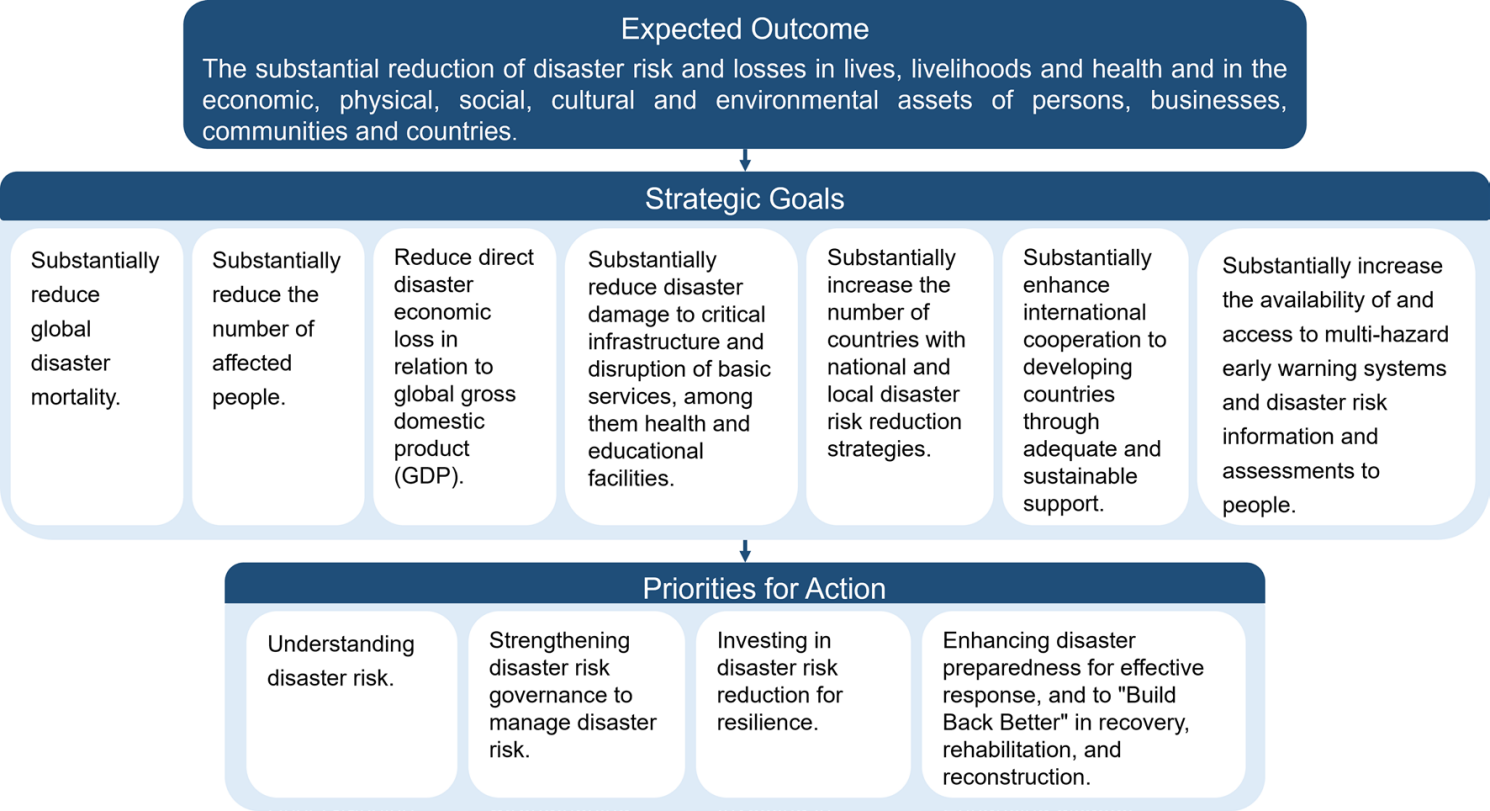
The Sendai Framework for Disaster Risk Reduction 2015−2030: Expected outcome, strategic goals, and priorities for action (United Nations 2015a)

In March 2015, the Third World Conference on Natural Disaster Reduction held in Sendai, Japan, adopted the Sendai Framework for Disaster Risk Reduction 2015−2030 (United Nations 2015a). The Sendai Framework set out an expected outcome and seven quantitative goals to be achieved in the following 15 years, together with four priorities for actions—understanding disaster risk, strengthening disaster risk governance to manage disaster risk, investing in DRR for resilience, and enhancing disaster preparedness for effective response and to “Build Back Better” in recovery, rehabilitation, and reconstruction (Fig. 3). The endorsement of the Sendai Framework opened a new chapter for DRR and sustainable development.

**Fig. 4:**
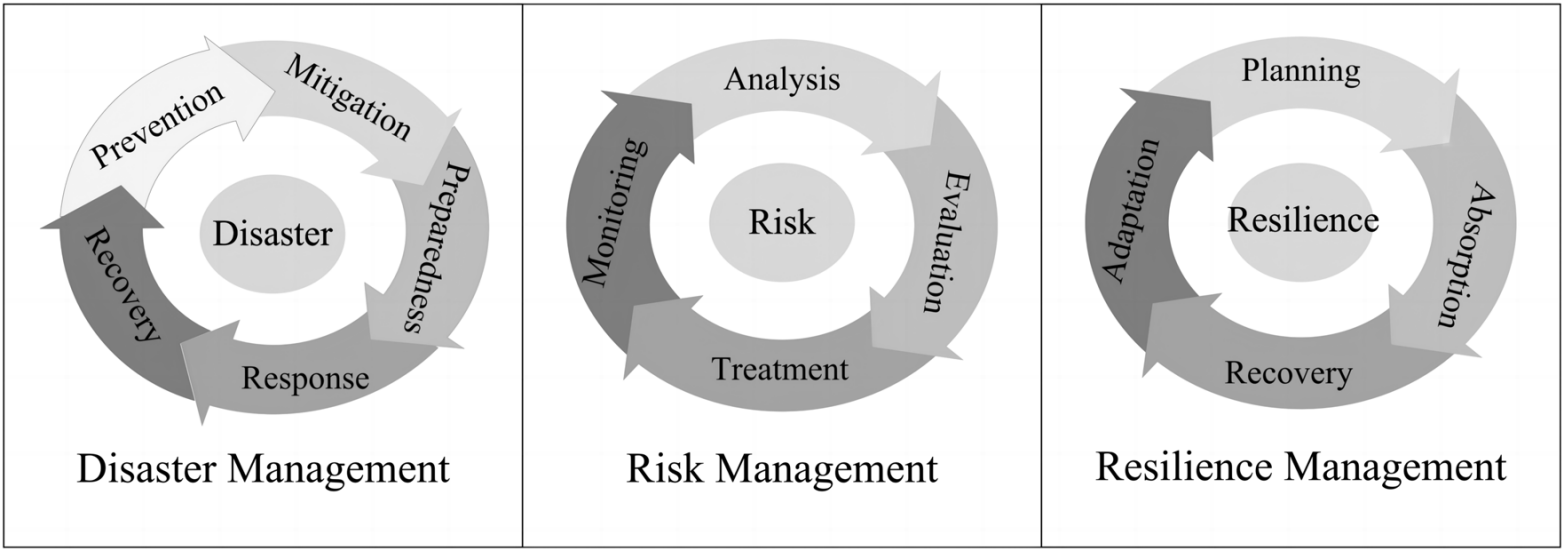
A comparison between disaster management, risk management, and resilience management

Over the past 30 years, in general, the development of DRR and related goals and priorities for action can be divided into three stages of disaster management in the 1990s, disaster risk management in the 2000s, and resilience management and development in the 2010s. The three stages reflect the key characteristics and important conceptual development of DRR actions at different stages rather than being separated from each other. Disaster management focuses on disaster-centered approaches (Fig. 4), and countermeasures are focused on disaster preparedness and response. Disaster risk management is to prevent new disaster risk, reduce existing disaster risk, and manage residual risk on the basis of risk-based decisions. It emphasizes risk-centered approaches (Fig. 4), and prevention and reduction are superior to response and relief. Resilience management (Fig. 4) is a new paradigm, which puts the emphasis on enhancing the ability of a system, community, or society to resist, absorb, accommodate, adapt to, transform, and recover from the effects of a hazard (predictable or unpredictable) in a timely and efficient manner, including through the preservation and restoration of its essential basic structures and functions through risk management.^3^ These ideas are embodied in the three World Conferences on Natural Disaster Reduction held by the United Nations and the adopted disaster risk reduction strategies and action frameworks.

The 1990s coincided with the IDNDR, which emphasized the enhancement of national disaster management capabilities in disaster prevention, mitigation, preparedness, and relief. The Yokohama Strategy urged the enhancement of disaster management for achieving sustainable development, and clarified that to achieve the goals of the IDNDR, disaster prevention, mitigation, and preparedness were more effective than disaster relief (IDNDR 1994). The 2000s witnessed the transition from disaster management to risk management. The Hyogo Framework emphasized that the focus of DRR should shift to disaster risk management and that DRR should be a national and a local priority and incorporated into national development policies (United Nations 2005). In the 2010s, the concept of the DRR field further shifted to resilience building. Researchers and practitioners at different levels worked a lot on the theory and practice of resilience, and gradually resilient management and development became an international consensus (Cutter et al. 2013; Florin and Linkov 2016; Gencer 2017).

### Climate Change Risk Assessment and Adaptation

Over the past 30 years, the IPCC has issued a series of comprehensive assessment reports about the state of scientific, technical, and socioeconomic knowledge on climate change impacts, risks, and adaptation. The adaptation negotiations under the UNFCCC have also made significant progress, and gradually, CCA has been widely implemented to overcome the adverse effects of climate change at all levels.

#### The Intergovernmental Panel on Climate Change Reports

Since 1988, every 6−7 years, nearly a thousand scientists around the world have engaged in various fields of climate change and socioeconomic and sustainable development to provide policymakers with a comprehensive explanation of the current international scientific community’s latest understanding of climate system changes in so far six assessment reports (see Fig. 1). Since 1990, IPCC’s six climate change assessment reports have made fruitful evaluations of the scientific progress of climate system changes, the impacts and risks of climate change on natural and socioeconomic systems, and the options for limiting greenhouse gas emissions and mitigating climate change. The reports have become authoritative documents for the international community’s combat of climate change, providing a scientific basis for the negotiations of the UNFCCC, and an important scientific basis for governments to formulate policies and take actions on climate change mitigation and adaptation (Qin 2018). In order to assess the relationship between climate change and extreme weather events, and their impacts on the sustainable development of society, the IPCC issued a special report on “Managing the Risks of Extreme Events and Disasters to Advance Climate Change Adaptation” in February 2012 (IPCC 2012). The report pointed out that the extent of damage caused by extreme weather to elements at risk depends not only on the extreme events, but also on the exposure and vulnerability of the social-ecological systems. The report also systematically explains the paths and methods of disaster risk management to adapt to climate change.

Adaptation is an important part of the IPCC reports. The IPCC Fifth Assessment Report (AR5) summarizes the adaptation needs, options, plans, and measures of climate change, and assesses the role of adaptation, the limitations of adaptation, and the transformation of adaptation in four chapters. The report gives a variety of adaptation measures, which can be grouped into three categories—measures to reduce exposure, incremental adaptation measures, and transformational adaptation measures (IPCC 2014). The IPCC Sixth Assessment Report (AR6) Working Group II (WGII) report describes the current status of adaptation and its benefit, future adaptation options and their feasibility, adaptation limitations, and maladaptation and how to avoid it. The feasibility of 23 adaptation measures is evaluated, which shows adaptation is subject to hard and soft limits (IPCC 2022).

#### Adaptation Negotiations Under the United Nations Framework Convention on Climate Change

Damage and loss associated with climate change impacts have emerged as key issues underpinning climate change adaptation at the global level during recent climate change negotiations under the UNFCCC (Prabhakar et al. 2015). Along with the rise in climate-related hazards, and the impacts and risks of fast-onset extremes and slow-onset changes (such as sea level rise) in the climate system, adaptation started attracting more attention at COP 10 (Conference of the Parties in 2004), then received successive boosts from the adoption of the Bali Action Plan in 2007 and the following COPs in Cancun (Mexico) in 2010 and others leading up to the 2015 Paris Agreement (Shaw et al. 2016) (see Fig. 1).

In December 2015, the Paris Climate Change Conference reached a series of results centered on the Paris Agreement, which became an important historical and binding international framework aiming to strengthen the global response to the threat of climate change (United Nations 2015b).The Paris Agreement puts forward three goals:

1. Holding the increase in the global average temperature to well below 2 °C above pre-industrial levels and striving to limit the temperature increase to 1.5 °C above the pre-industrial levels;
2. Increasing the ability to adapt to the adverse impacts of climate change and foster climate resilience and low greenhouse gas emissions development, in a manner that does not threaten food production; and
3. Making finance flows consistent with a pathway towards low greenhouse gas emissions and climate-resilient development.

In terms of adaptation and reduction of the damage and loss caused by climate change, global adaptation goals have been proposed to enhance adaptability, strengthen resilience, and reduce vulnerability to climate change.

Over the past 30 years, the adaptation negotiations under the UNFCCC can be roughly divided into three stages of early slow progress, equal emphasis on adaptation and mitigation, and enhanced adaptation action. The climate negotiations were characterized by “emphasis on mitigation, neglect of adaptation” in the early stage. After the 2007 Bali Roadmap adopted by the 13th Conference of the Parties (COP 13) that put equal emphasis on mitigation and adaptation, the adaptation-related agenda and its importance were increased under the UNFCCC negotiation regime. The 2010 Cancun Adaptation Framework and the 2015 Paris Agreement put forward specific action frameworks to enhance global adaptation actions, and to establish an international governance and mechanism for global adaptation to climate change, which laid a good foundation for enhancing climate resilience, reducing vulnerability, and achieving the goals of the UNFCCC (Tao 2014; Chen et al. 2016; Chen 2020).

### Linkages of Disaster Risk Reduction, Climate Change Adaptation, and the Sustainable Development Goals

In 1987, the Report of the World Commission on Environment and Development “Our Common Future” put forward the strategy of sustainable development, marking the birth of a new concept of development (WCED 1987). In June 1992, the United Nations Conference on Environment and Development (also known as the Earth Summit) adopted a series of important documents—the Rio Declaration on Environment and Development (also known as the Earth Charter); Agenda 21; the Framework Convention on Climate Change; and the Convention on Biological Diversity. The United Nations Convention to Combat Desertification was adopted on 17 June 1994. The Earth Summit established a road map of sustainable development with harmonious coexistence between humans and nature (United Nations 1992b; Cicin-Sain 1996). A considerable incentive for rethinking disaster risk as an integral part of the development process comes from the aim of achieving the goals laid out in the Millennium Declaration. The Declaration sets forth a road map for human development supported by 191 nations in 2000 (UNDP 2004). Following the end of the 2000−2015 Millennium Development Goals (United Nations 2000), the United Nations Development Summit in September 2015 unanimously adopted the draft resolution “Transforming our world: The 2030 Agenda for Sustainable Development,” submitted by the 69th session of the United Nations General Assembly (United Nations 2015c). The SDGs in the United Nations 2030 Agenda replaced the Millennium Development Goals launched by the United Nations at the beginning of the 21st century.

The agenda includes 17 SDGs and 169 associated targets. These development goals all closely interact and influence climate change and disaster risks. For example, Goal 9 building resilient infrastructure, Goal 11 building inclusive, safe, resilient, and sustainable cities and human settlements, and Goal 13 taking urgent action to combat climate change and its impacts, all are directly related to DRR and CCA. Many of these 169 associated targets also involve reducing disaster risks and disaster impacts. For example, one of the specific targets of Goal 1 is to build the resilience of the poor and those in vulnerable situations and reduce their exposure and vulnerability to climate-related extreme events and other economic, social, and environmental shocks and disasters by 2030. Disasters put development at risk, and losses caused by climate change and extreme events may severely hinder many countries from achieving SDGs. At the same time, the realization of the SDGs will also help reduce human vulnerability to climate change and disasters, thereby greatly reducing disaster risks.

**Fig. 5:**
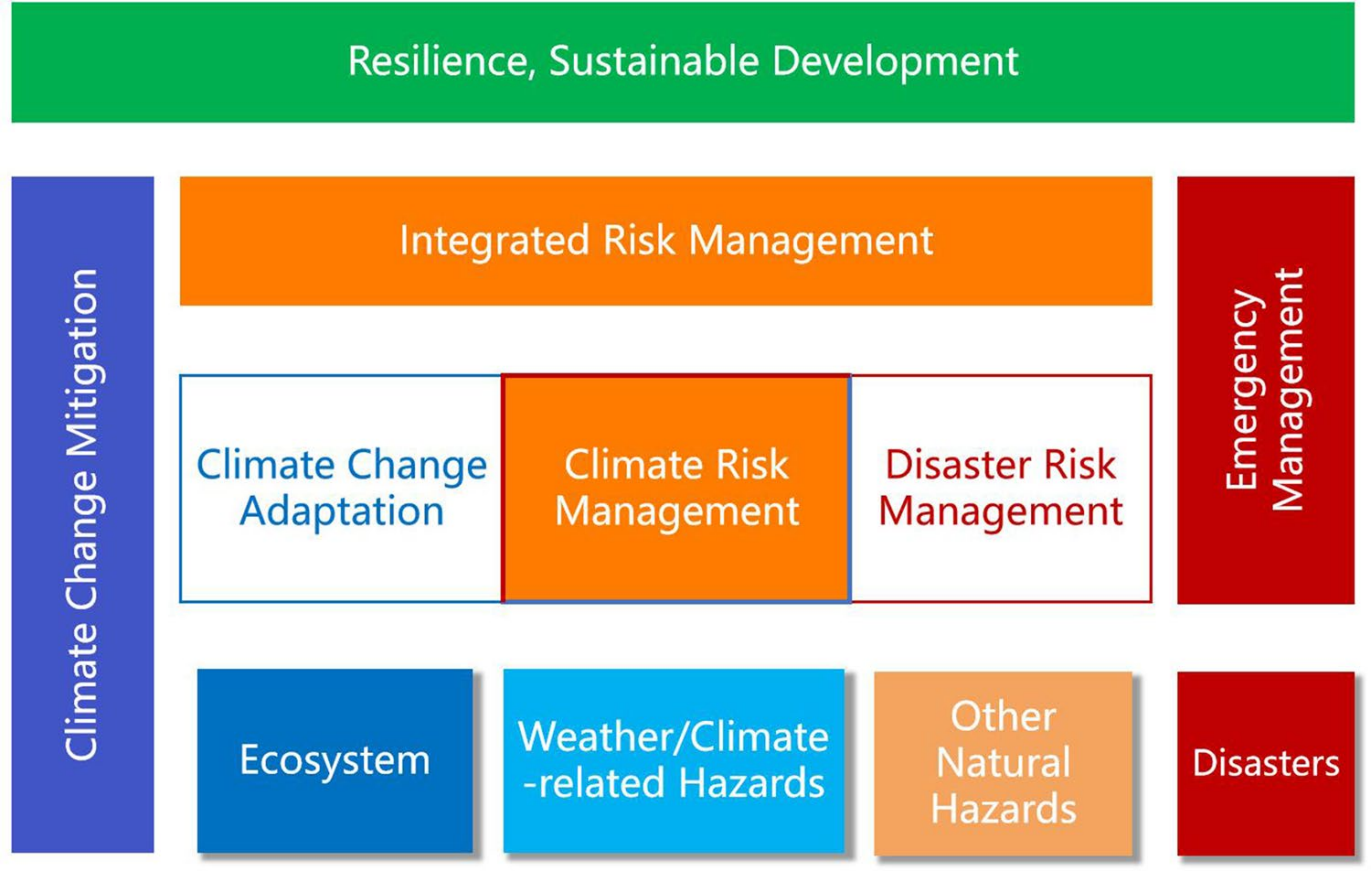
A framework for addressing disaster and climate change risks in the context of resilient, sustainable development

Climate change adaptation and DRR have similarities and differences in their scope and emphasis (Twigg 2015; Clegg et al. 2019). The common aim of CCA and DRR is to manage the risk induced by weather/climate-related hazards, including extreme events and climate-related creeping environmental changes, which is part of climate risk management (see Fig. 4). Their difference is that DRR not only deals with hydrometeorological disaster risk closely related to climate change, but also manages risks caused by other natural hazards, such as earthquakes and volcanic eruptions (Twigg 2015). In addition, DRR focuses more on reducing the potential losses of people and assets. Climate change adaptation also has its focus areas, such as the impact of climate change on ecosystems and biodiversity, and infectious diseases and health (IPCC 2022). According to the Adaptation Gap Report 2022 (UNEP 2022), CCA actions are currently mainly focused on agriculture, water, ecosystems, and cross-cutting sectors. Disaster risk reduction and CCA are two major areas of integrated risk management (Fig. 5), thus both should be joined within the integrated risk management that is an important pillar and field of resilient, sustainable development. Under the framework of resilient development, there are two areas that are closely related to climate change and DRR, that is, emergency management and climate change mitigation (Fig. 5). The synergistic effects of integrated risk management, emergency management, and climate change mitigation will effectively ensure safe growth and resilient development.

## Discussion

In many ways, DRR and CCA have overlapping aims and involve similar kinds of intervention (Begum et al. 2014; Forino et al. 2015; Twigg 2015; Amaratunga et al. 2017).

People and ecosystems across the world are already confronted with limits to adaptation, and if the planet warms beyond 1.5 °C or even 2 °C, more widespread breaching of adaptation limits is expected (Forino et al. 2015; Twigg 2015).

Addressing climate change may have the potential to create or exacerbate other development concerns (Kelman et al. 2015). Large dams might contribute to climate change mitigation and adaptation through reduced dependence on fossil fuels and regulating floods. But large dams tend to increase flood risk over the long term in a process termed ‘‘risk transference’’ (Etkin 1999). Seawalls and infrastructural development along coastlines may also induce changes in water currents, destruction of natural ecosystems, and increased or shifted erosion from protected to unprotected areas (Dahl et al. 2017; Rahman and Hickey 2019; Piggott-Mckellar et al. 2020; Simon et al. 2020). Seawalls may effectively reduce impacts to people and assets in the short term but may also result in lock-ins and increase exposure to coastal hazards in the long term unless they are integrated into a long-term climate risk management plan. Although fire suppression in naturally fire-adapted ecosystems prevents fire damage, such action reduces the space for natural processes, thus reducing the ecosystem’s resistance to climate change and its ecosystem service value (Ruffault and Mouillot 2015; Hope et al. 2016).

Therefore, DRR and CCA should be addressed together under integrated risk management to overcome limits and maladaptation, and optimize the use of limited resources (Mitchell et al. 2010; Twigg 2015; Flood et al. 2022). Thus, the integration of CCA and DRR can contribute to achieving the goals of international frameworks such as the SDGs (Kelman and Gaillard 2010; UN DESA 2014; Kelman 2017; Clegg et al. 2019), the Sendai Framework, and the Paris Agreement (Amaratunga et al. 2017).

**Fig. 6:**
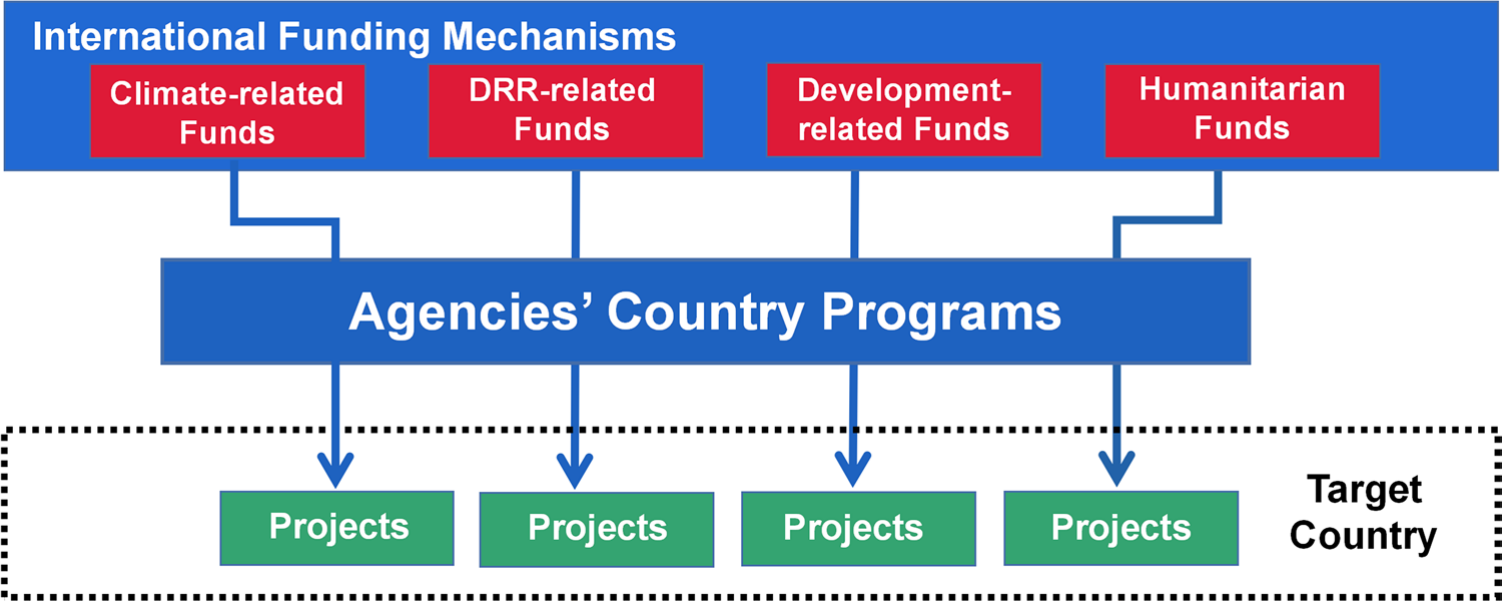
A scheme showing international funding mechanisms for target countries

However, there are many factors that hinder successful integration of CCA and DRR (Amaratunga et al. 2017; Seidler et al. 2018; Dias et al. 2020; Islam et al. 2020). Barriers include poor communication between organizations, coordination challenges, lack of political willingness, lack of capacity among actors and institutions, policy gaps, mismatches, different funding systems, fund shortages, and so on. Disaster risk reduction and CCA are frequently addressed, studied, and analyzed independently (O’Brien and Li 2006; Ireland 2010; Kelman et al. 2015; Chmutina et al. 2016; Clegg et al. 2019), separated by institutional and administrative boundaries (Schipper and Pelling 2006; Kelman 2017; Pilli-Sihvola 2020). For historical and political reasons, internationally, the way we are currently working addresses climate change, DRR, development-related projects, and humanitarian relief separately (Fig. 6). International funding mechanisms establish and implement independent projects of CCA, DRR, and so on in target countries through international organizations (such as different agencies of the United Nations), resulting in segmented practices.

At the national level, CCA and DRR are also frequently handled independently, separated by institutional and administrative boundaries (Schipper and Pelling 2006; Kelman 2017; Dias et al. 2018; Clegg et al. 2019). In China, for example, the Fourteenth Five Year Plan for National Comprehensive Disaster Prevention and Reduction (2021−2025) was formulated by the National Disaster Reduction Commission, which is only a deliberative body and thus it is difficult to promote the implementation of the plan. In 2022, 17 national departments jointly issued the National Climate Change Adaptation Strategy 2035, with the Ministry of Ecology and Environment as the leading department. Climate change adaptation and DRR efforts are still addressed by two sets of organizations in China. In the Philippines, DRR and CCA are operationalized independently of one another (Florano 2015; De Leon and Pittock 2017). There are two separate laws on climate change and disaster risk reduction and management—the Climate Change Act of 2009 and the Philippine National Disaster Risk Reduction and Management Act of 2010, respectively. This is also the case in national level arrangements in the UK, where DRR and CCA are managed by separate government departments (Dias et al. 2018; Clegg et al. 2019).

**Fig. 7:**
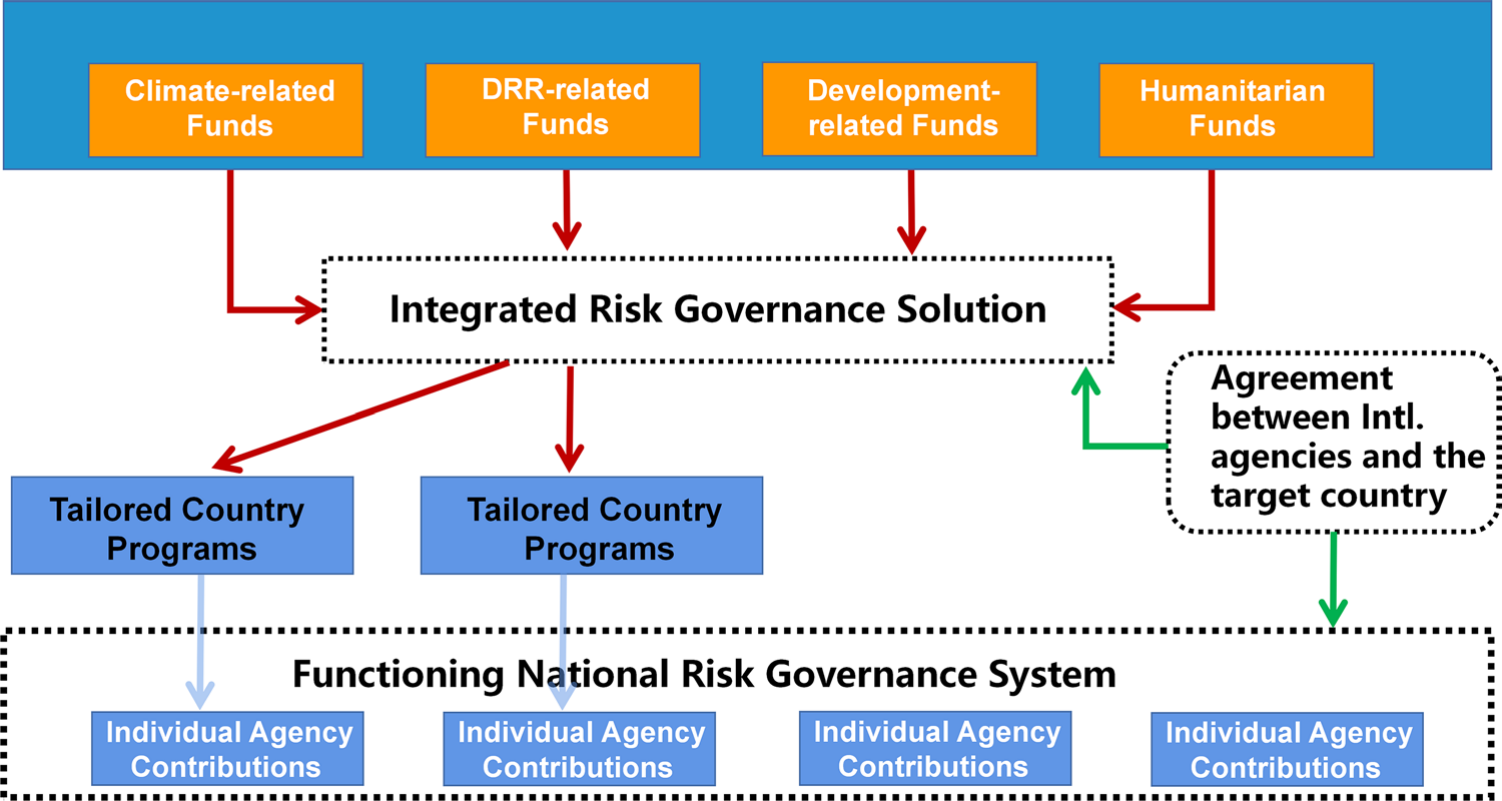
Integrated risk governance solution among international organizations and countries

To change this situation, effective governance mechanisms, such as policy, agreement, culture, leadership, and coordination need to be established among international organizations, as well as between international organizations and target countries, while countries also need to establish overarching national risk governance systems (Fig. 7). Thus, tailored country programs can be established through international risk governance solutions, and implemented in target countries by a unified mechanism under the national risk governance system.

Moreover, a wide range of climate change impacts and disaster risks (especially the cascading and systemic risks) are understudied or challenging to quantify, and are missing from current evaluations of climate change and other disaster risks to lives and assets (Mamuji and Etkin 2019; Mcglade et al. 2019; Rising et al. 2022). Importantly, integrated risk and resilience management is about managing known risks but also about preparing for the unpredictable (Pirani and Tolkoff 2015). Thus, better data, actionable information, and relevant knowledge on climate change and disaster risk are needed to promote the integration of CCA and DRR (Mysiak et al. 2018; Zuccaro et al. 2020).

## Conclusion

This study reviews the major impacts and challenges of disaster and climate change risks on sustainable development, summarizes the important events and evolution of international disaster risk reduction and climate change adaptation over the past 30 years, and reviews the linkages of DRR and CCA to sustainable development. The three main conclusions are:

1. Disasters caused by both intensive and extensive disaster risks have a huge impact on lives and livelihoods. Indirect losses and cascading effects may cause even more serious damage to the socioeconomic development of a region or a society. Most disasters triggered by natural hazards are related to weather/climate events. Especially under a changing climate, compound events and systemic risks are increasing, and record-shattering extremes are likely to occur in the coming decades, which will significantly limit our ability to adapt.
2. Over the past 30 years, the evolution of paradigms in DRR actions can be roughly divided into three stages—disaster management in the 1990s, disaster risk management in the 2000s, and resilient management and development in the 2010s. These ideas are embodied in the three World Conferences on Natural Disaster Reduction held by the United Nations and the adopted disaster reduction strategies and action frameworks. The adaptation negotiations under the UNFCCC over the past 30 years also can be roughly divided into three stages of early slow progress, equal emphasis on adaptation and mitigation, and enhanced adaptation action. Climate change adaptation has been widely carried out to overcome the adverse effects of climate change. The integrated risk management community has also learned the current status of adaptation and its benefit, future adaptation options and their feasibility, adaptation limitations, and maladaptation and how to avoid it.
3. This article proposes a framework for addressing climate change and disaster risks in the context of resilient, sustainable development. Climate change adaptation is not a subset of DRR, and they have both similarities and differences in their scope and emphasis. Disaster risk reduction and CCA should be joined under the integrated risk management that is an important pillar of resilient and sustainable development. Under the umbrella of resilient development, there are two areas that are closely related to climate change and DRR—disaster management and climate change mitigation. The synergistic effects of integrated risk management, emergency management, and climate change mitigation will effectively support safe growth and resilient development.

To successfully integrate CCA and DRR, it is urgently needed to transform governance mechanisms, and to strengthen cooperation among international organizations, as well as between international organizations and countries, while countries also need to establish overarching national risk governance systems. Moreover, better data, actionable information, and relevant knowledge are needed for understanding climate change and disaster risks in a context of deep uncertainty.

The severe effects of the COVID-19 pandemic on our health and socioeconomic well-being are a stark warning of the dangers of insufficient actions, prevention, and preparedness—but people and societies can adopt new behaviors when the problems and situations are changing. In the context of climate emergency, the feasibility and effectiveness of adaptation measures will decrease with increasing warming. It is urgently needed to leverage the synergies of CCA and DRR, together with climate change mitigation and disaster management, in order to prevent new risks, reduce and mitigate existing vulnerabilities and risks, and to realize the goals of the Sendai Framework, the Paris Agreement, and the Sustainable Development Goals.

## Footnotes

1 https://www.ipcc.ch/about/history/.

2 https://www.undrr.org/about-undrr/history.

3 https://www.undrr.org/terminology/resilience.
